# Evolutionary Significance of the Neuroendocrine Stress Axis on Vertebrate Immunity and the Influence of the Microbiome on Early-Life Stress Regulation and Health Outcomes

**DOI:** 10.3389/fmicb.2021.634539

**Published:** 2021-04-07

**Authors:** Van A. Ortega, Emily M. Mercer, Gerald F. Giesbrecht, Marie-Claire Arrieta

**Affiliations:** ^1^Department of Physiology and Pharmacology, University of Calgary, Calgary, AB, Canada; ^2^International Microbiome Centre, Cumming School of Medicine, Health Sciences Centre, University of Calgary, Calgary, AB, Canada; ^3^Department of Pediatrics, University of Calgary, Calgary, AB, Canada; ^4^Department of Community Health Sciences, University of Calgary, Calgary, AB, Canada; ^5^Owerko Centre, The Alberta Children’s Hospital Research Institute, Calgary, AB, Canada

**Keywords:** cortisol, inflammation, gut-brain axes, HPA axis, immunity, pediatrics, vertebrate evolution, physiology

## Abstract

Stress is broadly defined as the non-specific biological response to changes in homeostatic demands and is mediated by the evolutionarily conserved neuroendocrine networks of the hypothalamus-pituitary-adrenal (HPA) axis and the sympathetic nervous system. Activation of these networks results in transient release of glucocorticoids (cortisol) and catecholamines (epinephrine) into circulation, as well as activation of sympathetic fibers innervating end organs. These interventions thus regulate numerous physiological processes, including energy metabolism, cardiovascular physiology, and immunity, thereby adapting to cope with the perceived stressors. The developmental trajectory of the stress-axis is influenced by a number of factors, including the gut microbiome, which is the community of microbes that colonizes the gastrointestinal tract immediately following birth. The gut microbiome communicates with the brain through the production of metabolites and microbially derived signals, which are essential to human stress response network development. Ecological perturbations to the gut microbiome during early life may result in the alteration of signals implicated in developmental programming during this critical window, predisposing individuals to numerous diseases later in life. The vulnerability of stress response networks to maladaptive development has been exemplified through animal models determining a causal role for gut microbial ecosystems in HPA axis activity, stress reactivity, and brain development. In this review, we explore the evolutionary significance of the stress-axis system for health maintenance and review recent findings that connect early-life microbiome disturbances to alterations in the development of stress response networks.

## Introduction

William Shakespeare’s “The Tempest” is famed for its literary citation, *“What is past is prologue*,*”* suggesting that the context of the present is determined by the precedents of the past. This allegory has often been applied to societal and cultural politics; however, it further extends relevancy to that of biological life and the maturation of its complex and multifaceted physiological systems. The developmental origins of diseases are often best viewed using an evolutionary lens to examine the underpinnings of when the affected physiological systems originated, as well as how and why they have been adaptively selected. Understanding biological systems from their inception provides insights into malfunctions that have occurred under modern environmental conditions.

From an evolutionary perspective, physiological stress response systems have always been indispensable for organisms to appropriately evaluate the stochastic or unpredictable aspects of their environments and adapt accordingly to maintain homeostasis and ensure their survival. Therefore, the broad concepts of stress and homeostasis are interwoven, whereby homeostasis is the maintenance of relatively stable internal bodily compartments in the face of changing external conditions by using feedback mechanisms to vary internal activities and minimize deviations from established physiological set points. Stress, by contrast, perturbs homeostasis, and stress responses are the physiological cascade of events that occurs when an organism attempts to re-establish homeostatic norms in the face of perceived threats. The stress response, therefore, has clear and fundamental adaptive advantages, and evidence has shown the molecules and peptides that regulate physiological responses to stress have remained remarkably conserved for over 500 million years of vertebrate evolution (Lovejoy et al., 2014).

Similarly, immunity has existed for hundreds of millions of years as a vital physiological system that protects the host from internal and external dangers to infections and changes in homeostasis (Plouffe et al., 2005). Therefore, both stress and immune responses have fundamentally evolved as defense systems (Burges Watson et al., 2016), with evidence suggesting they likely co-evolved from a common origin (Ottaviani, 2011). Molecular trade-offs from a common pool of molecules have created deep phylogenetic interactions between the neuroendocrine and immune systems that help explain their continual bilateral integration and responses to environmental stressors (Ottaviani, 2011).

**FIGURE 1:**
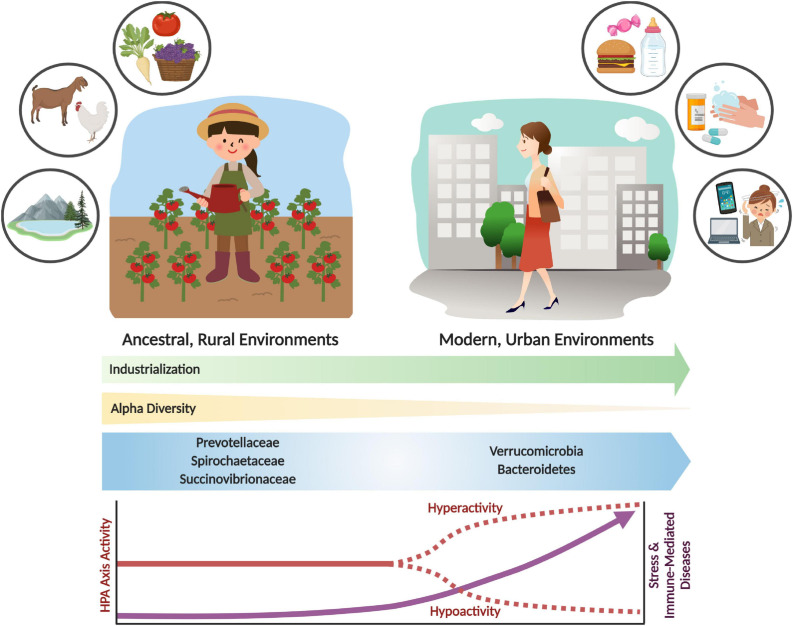
Shifts in the gut microbiome and stress response activity with industrialization and urbanization. Urbanization and industrialization have transformed environmental and microbial communities in modern environments. This has resulted in shifts to gut microbial composition, decreased alpha diversity, and loss of key microbial taxa (e.g., Prevotellaceae, Spirochaetaceae and Succinovibrioaceae families). These changes may correlate with divergence from ancestral environments and lifestyles, which includes rural habitation, whole food diets, and increased exposure to environmental microbes and antigens. Modern industrialization provides increased environmental and personal sanitization, pharmaceutical and antibiotic use, exposure to psychological stressors, and consumption of processed foods. These lifestyle changes have had significant impacts on the microbiome and on stress physiology, which can result in stress and immune-related diseases. The parallels between environmental, microbiome and disease incidence shifts are likely not coincidental. Rather, an evolutionary mismatch has led to adaptive responses becoming maladaptive, resulting in adverse shifts in lifelong health trajectories.

However, despite the adaptive utility of acute stress and immune responses (Ottaviani, 2011; Brenner et al., 2015; Nesse et al., 2016), chronic activation can harm the host and result in various disease states (Brenner et al., 2015). Evolved traits that were once advantageous to an organism can become dysfunctional in different environments (Parker and Ollerton, 2013). This is the basic concept of evolutionary mismatch, which offers insight into the modern industrialized environmental conditions that trigger contemporary psychological and immune-related diseases, which were seemingly less prevalent in ancestral environments (Brenner et al., 2015; Figure 1).

An evolutionary mismatch that has gained attention in the past decade is the loss of diversity and keystone species from the communities of microbes in the human gut (the microbiota) and their genetic material (the microbiome), which is hypothesized to be at the root of the increased psychological- (e.g., anxiety, depression) and immune- (e.g., allergies, asthma, inflammatory bowel diseases) related chronic diseases (Bilbo et al., 2011; Brenner et al., 2015). The ever-present symbiotic partnership between humans and microbes has brought significant benefits to human evolution, physiology, and health. The loss of these “old friends” and their associated functions (i.e., microbiome depletion) (Rook et al., 2003) with developments to modern society such as sanitation, water quality, antibiotic use, Cesarean (C-) sections, diet, and other factors, termed the microbiome insufficiency syndrome (Sonnenburg and Sonnenburg, 2019), has likely had significant effects on the prevalence of modern diseases (Cryan et al., 2019; Figure 1). In this review, we will examine the evolutionary trajectory and integration of the stress and immune systems in vertebrates to gain a fundamental appreciation of their critical role in animal adaptive fitness. This initial evolutionary assessment will set the stage for the role microbes have had in priming these systems to function as efficient defenders of homeostasis and infection. Finally, this review will examine the consequences of microbiome alterations on stress systems and health and disease outcomes.

## The HPA Stress Axis

All organisms, including humans, continually encounter internal and external changes in the environment (e.g., infections or predatory threats) which may trigger a stress response that overturns their internal rhythms and homeostasis. Sufficient deviations from established set points constitute a “stress” to the organism, which are addressed by various bodily responses to allow the animal to overcome the threat and re-establish homeostasis. Thus, the concept of stress is closely related to the concept of homeostasis, whereby the latter represents an established optimal state for key biological parameters, while the former is a state that arises when homeostasis is disrupted (Monaghan and Spencer, 2014).

Perception of a stressor (conscious or not) is communicated to the body via the central nervous system (CNS), which acts both neuronally for an immediate reaction, and hormonally for a delayed, prolonged response (Burges Watson et al., 2016). Therefore, a stress can be broadly defined as a series of events that starts with a stimulus (i.e., the stressor), which triggers a reaction (i.e., perception of stressor), and results in the activation of various physiological systems (i.e., stress response) (Harris and Carr, 2016). The activation of the stress response is dependent on individual evaluative coping capacities, whereby physiological responses can be blunted if the capacity to cope with the stressor is effective. Nevertheless, according to Hans Selye’s original general adaptive syndrome (GAD) theory for coping with stress (Selye, 1950), any disturbance to an organism’s homeostasis can initiate an initial rapid response, or “alarm phase,” constituting the perception of the stimulus and recognition of a potential homeostatic threat. If the effort to deal with the stressor is very large, or coping is low, a “resistance stage” follows, in which the organism mobilizes energy stores to restore homeostasis. This is known as allostasis, and the physiological effort to do so is termed the allostatic load (Monaghan and Spencer, 2014). Finally, if the disturbance is longer lasting or overly intense, the organism would enter the “stage of exhaustion,” in which it may lose the capacity to cope with the disturbance, leading to adverse health outcomes. Accordingly, these response stages have been broadly categorized into primary, secondary, and tertiary levels.

Primary responses involve two biochemical axes that elicit cellular changes in the adrenal gland when a “threat” is perceived - one through the fast-acting sympathetic nervous system (SNS) and the other through the slow-acting hypothalamic-pituitary-adrenal (HPA) axis. Both axes of the primary response are readily activated, and their actions are designed to prioritize vital bodily functions that prepare the organism for rapid engagement against the stressor (Nesse et al., 2016).

Following perceived threats, SNS responses are immediately propelled through efferent sympathetic nerve fibers that result in the circulatory release of catecholamines (epinephrine (EPI) and norepinephrine (NE)) from innervated cholinergic receptors on chromaffin cells in the adrenal medulla. EPI and NE are also immediately released from fibers that directly innervate other bodily organs, such as the heart. The release of catecholamines directly into tissues and circulation have immediate and potent effects on respiration and cardiovascular systems (Randall and Ferry, 1992), blood oxygen transport capacity (Nikinmaa, 1992), and blood glucose and free fatty acid levels (Van Raaij et al., 1995) that are collectively known as “fight or flight” responses.

The HPA axis is the second primary stress response axis, and involves the delayed release of glucocorticoids (GC) (e.g., cortisol or corticosterone in some mammals) from the adrenal cortex. Following a stressful stimulus, the hypothalamus responds to elevated NE by secreting corticotropin-releasing hormone (CRH) from neurosecretory neurons in the paraventricular nucleus (PVN) of the hypothalamus to influence the rapid release of adrenocorticotropic hormone (ACTH) from the anterior pituitary into circulation (Vale et al., 1981). ACTH then induces GC synthesis and release from the adrenal glands (Wendelaar Bonga, 1997), which circulate and bind to mineralocorticoid (MR) and glucocorticoid receptors (GR) on several peripheral and central tissues, including corticolimbic brain regions. Activated GR and MR modify a broad array of physiological functions that help the organism manage the stressor, including increasing metabolism (Vegiopoulos and Herzig, 2007; Timmermans et al., 2019), mobilizing glucose energy stores from the liver (Garabedian et al., 2017), and heightening arousal, focus and memory (Farrell and O’Keane, 2016).

Secondary responses involve changes in plasma and tissue ion and metabolite levels, modulation of immune responses, and general physiological adjustments (Barton, 2002), while tertiary responses involve whole animal changes, including growth rate, body condition indices, resistance to disease, and ultimately, survival (Burges Watson et al., 2016). GCs also regulate the potency of the stress response by exerting a negative feedback on the expression of CRH in the hypothalamus, and the release of ACTH at the pituitary to terminate the stress response and return the organism to homeostasis (Brunson et al., 2001).

The categorization of stress into these defined levels is dependent on many factors, including the type or intensity of a stressor, the age and sex of an individual, as well as the organism’s own perception of the threat to its homeostasis (Harris and Carr, 2016; Nesse et al., 2016). For example, the HPA stress system is activated by external environmental stressors (i.e., physical threats like predators, weather, resource shortages), but also by psychological stressors (i.e., internal threats) that may not be an actualized danger, such as the recall of a stressful memory (Monaghan and Spencer, 2014). In small rodents that face predation, olfactory cues elicit the strongest activation of the HPA axis, followed by auditory, and lastly, visual cues (Harris and Carr, 2016), showing that HPA activity has a gradation of responses that can be tuned to a perceived threat.

Sex differences are another important factor that affects the HPA response to stress in both humans and animals. In general, HPA-related stress molecule levels are higher in female rodents and rise faster than in males following HPA axis stimulation (Yoshimura et al., 2003; Beiko et al., 2004; Kudielka and Kirschbaum, 2005; Lu et al., 2015). Female rats exposed to chronic and unpredictable mild stressors have disordered estrous cycles, decreased estradiol and testosterone levels, increased plasma corticosterone levels, and upregulated gene expression of both CRH and estrogen receptor β (ERβ) (Lu et al., 2015) in the hypothalamic neurons of the PVN. Direct actions by estradiol on the PVN can potentiate the stress axis by targeting neurons expressing ERβ to activate CRH promoters (Miller et al., 2004) and increase CRH gene expression and basal levels of ACTH (Ochedalski et al., 2007). Ovariectomy in females reduces these HPA hormones, while estradiol replacement treatment recovers them (Lund et al., 2004). Male rodents that are castrated have elevated corticosterone and ACTH levels similar to females, which is also reversed using exogenous androgen treatments (Handa et al., 1994). Empirical evidence for sex differences in humans is more equivocal than animal data (Kudielka and Kirschbaum, 2005). Physical stressors do not elicit responses that are different between males and females (Friedmann and Kindermann, 1989), and most psychological stress studies show either no difference, or that males have increased cortisol responses (Kudielka and Kirschbaum, 2005; Liu et al., 2017).

## Evolution of the Stress Axis in Vertebrates

**FIGURE 2:**
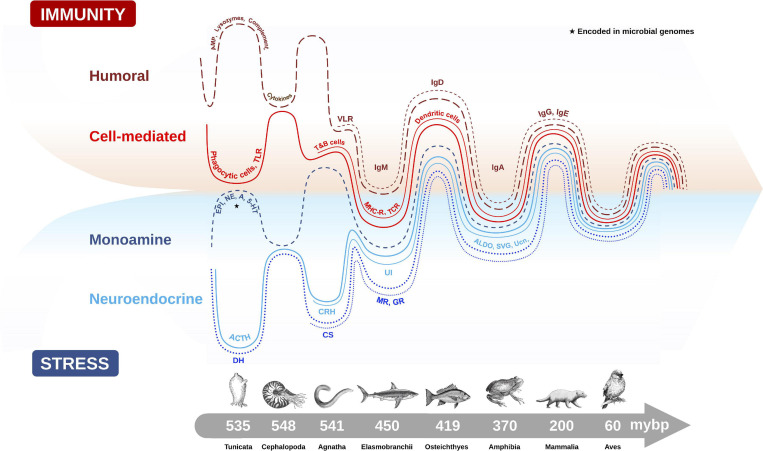
Evolutionary emergence and biological integration of key cellular and molecular features of the immune (humoral and cell-mediated) and stress axis (neuroendocrine and monoamine) systems in chordate animals. Converging timescale lines depict increasing complexity and integration between the two physiological systems as new classes of vertebrates arose over millions of years before present (mybp). Gut microbial communities also influence host physiology by directly producing biomolecules (e.g., monoamines) encoded in microbial genomes (★), or by influencing the production of host stress and immune molecules. The complex interaction between the microbiome and host plays an important role in regulating many host physiological processes, like metabolism, immunity and stress responses. Features of humoral immunity include: antimicrobial peptides (AMP), lysozymes, complement proteins, cytokines, and variable lymphocyte receptors (VLR), which function as antigen-binding antibodies in basal fish like jawless hagfish, and are precursors to immunoglobulins (Igs) (e.g., IgM, IgD, IgA, IgG, IgE). Cell-mediated immune features depicted are: phagocytic cells which provided early immune protection in invertebrates, T and B cells, toll-like receptors (TLR), major histocompatibility complex (MHC), T-cell receptor (TCR) and dendritic cells. Monoamine features of the stress axis include epinephrine (EPI), norepinephrine (NE), dopamine (DA) and serotonin (5-HT), while neuroendocrine stress axis molecules include adrenocorticotropin hormone (ACTH) homologs in invertebrates and diuretic hormones (DH), which were likely precursors to the corticotropin releasing hormone (CRH) family of peptides, including urotensin 1 (UI) in fish, sauvagine (SVG) in amphibians and urocortin (Ucn) in mammals. Corticosteroids (CS) and aldosterone (ALDO) are terminal hormones of the stress axis, which bind to glucocorticoid receptors (GR) and mineralocorticoid receptors (MR), respectively, found in various tissues in the body to regulate stress reactivity and immunity.

The origin of the vertebrate stress axis is centered on an ancient neuroendocrine circuit (i.e., the HPA axis) that has remained remarkably conserved over vertebrate evolution, despite the emergence of phylogenetically distinct classes of animals (e.g., fish, amphibians, reptiles, birds, mammals) occupying a vast range of habitats over that same period (Denver, 2009; Dores and Garcia, 2015). Its pivotal role in responding to various environmental and internal threats to homeostasis and fitness was likely a key reason for its conservation across species. It is believed that the ancestral endocrine system of the modern stress axis originated in marine organisms for the purpose of eliminating excessive sodium load from seawater (Perlman, 2013). Indeed, osmoregulation is considered one of the more ubiquitous types of environmental stressors in these ancestral marine animals (Lovejoy et al., 2014). The diuresis origin theory is supported from evidence that invertebrates, such as insects and marine worms, secrete ACTH with high structural similarity to mammals and possess diuretic hormones (DH) with remarkable homology to mammalian CRH (Nesse et al., 2016). Within Animalia, CNS molecules that regulate the cascading stress axis also share high sequence homology between classes, with differences seen in the glucocorticoid-producing tissues (adrenal gland in reptiles, birds and mammals; and interrenal cells in amphibians and fish) at the terminal point of stress axis activation (Dores and Garcia, 2015). The capacity to synthesize GCs was an important evolutionary step that occurred under the jawless vertebrates (i.e., cyclostomata like hagfish and lamprey) (Norris and Carr, 2013), and represented a transition in which the central and terminal components of the HPA axis were established (Denver, 2009). The appearance of GR and MR from ancestral corticosteroid receptors (CR) during a gene duplication event 450 million years ago (Thornton, 2001) also created two new receptor lineages with distinct endocrine roles for regulating the ancestral need for osmotic balance (MR preferentially binds aldosterone) and stress responses (GR preferentially binds GCs). Finally, the conserved distribution patterns of CRH and it analogs (urotensins, urocortins and sauvagine that are CRH homologs) within and around the vertebrate hypothalamus of amphibians, fish, birds, reptiles and mammals also supports the theory of positive selection on ancient genes that regulate an evolutionarily conserved HPA axis (Denver, 2009; Figure 2).

Recently, there is an increasing awareness that colonization with gut microbes also plays an important role in the appropriate early-life development and functioning of the HPA axis in host species (Sudo, 2014; Foster et al., 2017), and raises intriguing questions about the role host microbes have in the phylogeny of the HPA axis. The role of the microbiome as an evolutionary driver of neuronal development has been examined in the context of the social brain hypothesis (Dunbar, 1998), which suggests that increased socialization between individuals favors the transmission of commensal bacteria that produce metabolites involved in regulating genes associated with cognition and anxiety (Stilling et al., 2014; Davidson et al., 2018). An adaptive component to microbiome variation could function to optimize social behavior, cognition, immunity, and host stress responses (Davidson et al., 2018).

The precise mechanisms by which regions of the brain recognize microbial signals from the gut have only recently been investigated, with promising candidate pathways discussed in more detail in later sections of this review. However, on the whole, a clear understanding of the bi-directional communications that exist between the gut and the brain continues to elude researchers. Given the symbiotic history of microbes and vertebrates (Ley et al., 2008), it is not surprising that an essential relationship exists between the highly conserved vertebrate neuroendocrine stress axis system and intestinal microbial communities. This close association between organisms involves the ecological assemblage of a host and its symbiotic microbial species (i.e., microbiota), resulting in an ecological unit that self-supports common outcomes and contributes to the function of the whole via inter-kingdom signaling mechanisms, such as host receptors for microbial enzymes involved in metabolic pathways for catecholamines (Iyer et al., 2004; Li et al., 2012), among many other described microbiome-derived metabolic products.

## Evolution of Host-Associated Microbiomes

Understanding the complex relationship between hosts and microbiomes from an evolutionary perspective helps to illuminate the influence the microbiome has had on the physiology of vertebrate hosts (Ley et al., 2008; Sharpton, 2018) and vice-versa. The intricate coexistence between animals and symbiotic microbes likely started with ancestral invertebrates that harbored high percentages of microbial communities, as they do today (Pancer and Cooper, 2006). These microbial relationships were transmitted to vertebrate descendants that still rely on their microbiome to communicate with host immune and stress systems for optimizing their health (Pancer and Cooper, 2006). Recent efforts investigating the contribution of the microbiome to vertebrate evolution have found that the dominant drivers of gut microbiome diversity and composition are diet (David et al., 2014; Maurice et al., 2015; Hicks et al., 2018; Youngblut et al., 2019) and species phylogeny (Groussin et al., 2017; Youngblut et al., 2019). Examination of dietary strategies found that the guts of carnivores were most similar to free living bacterial communities amongst vertebrates, while microbes with plant fermenting capacities contributed most to the divergence of vertebrate gut microbiomes (Ley et al., 2008) and were the best predictors for co-phylogeny with their hosts. Ruminants have evolved specialized digestive organs to enhance gut microbial functionality that they rely upon for accessing nutrients from fibrous plant material. This functional capacity to increase host-access to low quality or difficult to digest energy sources (like woody and cellulose vegetation), could improve niche expansion into other environments that leads to speciation (Sharpton, 2018). Similarly, microbes that produce essential vitamins and other necessary nutrients could also remove selective pressures from the host to obtain them through diet and alter their evolutionary trajectory (Sharpton, 2018). However, little is known about the functions encoded by the microbiome in wild vertebrate populations, and this research gap should be addressed to understand how microbiome functions vary across vertebrates and dictate evolutionary outcomes (Sharpton, 2018). Nevertheless, recent studies have found that mammals in general, have the highest pattern of cophylogeny with microbial species, while fish have the lowest; likely a result of having more transient environmental microbes in their guts (Youngblut et al., 2019). Interestingly, the human microbiota was similar to omnivorous primates, despite the variable and often synthetic nature of the modern human diet (Ley et al., 2008).

Based on the findings of these and other related studies, it is thought that animals who harbor microbial communities with specific metabolic properties or functional abilities are better able to resist and respond to environmental perturbations (Sharpton, 2018). This would result in longer-lived, healthier hosts able to produce more offspring and better disseminate microorganisms to other hosts at the benefit of the greater microorganism metacommunity (Ley et al., 2008). Kinship and social behavior in animals are a suggested mechanism for accessing and transferring beneficial microorganisms (Lombardo, 2008), which would promote the co-evolution of both microbial communities and host alike (Ley et al., 2008).

The influence of the microbiome on the development of the adaptive immune system in vertebrates is another example where the host and microbiome mutually benefit. For example, adaptive immunity mediates tolerance of the gut microbiome through IgA by driving perpetual surface structure modification in microbes that ultimately results in reduced proinflammatory signaling by immune factors in the host, and maintains a non-inflammatory connection to commensal gut microbes (Peterson et al., 2007). Thus, a key role of the adaptive immune system could be to shape microbial community composition and support microbial diversity (Peterson et al., 2007; Ley et al., 2008). Selective pressures from the host in a nutrient-rich gut environment, with high cell densities and growth rates, is an ideal environment for rapid microbial evolution that is not always found in free-living ecosystems, which can be cold and oligotrophic (Ley et al., 2008).

## Integration of Stress Responses With the Immune System

**FIGURE 3:**
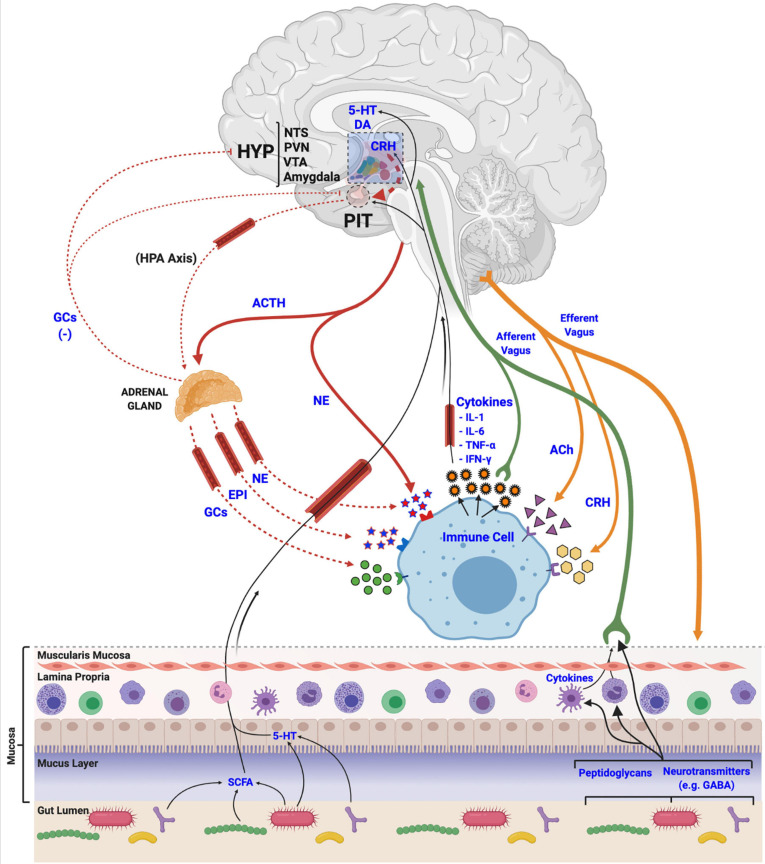
Mechanisms of stress axis and immune system interactions and biological integration. Activation of the hypothalamic-pituitary-adrenal (HPA) axis terminates with the release of glucocorticoids (GCs), epinephrine (EPI) and norepinephrine (NE) at peripheral tissues, like the adrenal gland, or directly from nerve endings. These stress-related molecules bind to receptors located in various tissues in the body to manage physiological responses to stress, and to receptors on immune cells and tissues to regulate immune and inflammatory processes, including the release of various cytokines. Released cytokines, in turn, travel via circulation and afferent fibers of the vagus nerve to the central nervous system to interact with various brain regions like the hypothalamus (HYP), the nucleus tractus solitarii (NTS), paraventricular nucleus (PVN), ventral tegmental area (VTA), and the amygdala, to regulate the activity of the stress axis. Acetylcholine (ACh) and corticotropin releasing hormone (CRH), released from efferent nerve fibers, also interact with immune cells to regulate functions. Finally, gut microbial communities (i.e., the microbiome) release various metabolic products (e.g., short chain fatty acids (SCFA), monoamines, neurotransmitters and other features, like peptidoglycans) that are utilized, incorporated and recognized by the host and its immune cells to regulate both stress and immune systems via afferent vagal nerve terminals.

The development of a robust HPA axis system has clear adaptive advantages for animals to manage various stressors. Coordination with other physiological systems is a critical aspect of the stress response to appropriately deploy necessary resources and efficiently maintain homeostasis (Martin, 2009). The well-defined actions of stress on metabolism, cardiovascular output, glucose transport, and immunity (Sapolsky et al., 2000) are orchestrated by innervating fibers of the SNS that target the gut and other primary and secondary organs of the immune system (e.g., bone marrow, thymus, spleen, and lymph nodes) (Martin, 2009), and circulating hormones like GCs that bind to GRs and MRs present on many visceral organs and immune cells (Thornton, 2001; Petta et al., 2016; Figure 3). Although this multi-systems approach to stress allows for phenotypic variability and adaptability in changing environmental conditions (Brenner et al., 2015), activating a broad array of physiological systems, like immunity, it can come at a long-term pathological cost to the animal if inflammatory and/or stress responses persist chronically or are not appropriately regulated (Brenner et al., 2015).

Vertebrate immune responses are broadly categorized into the innate (generalized and ancestral) and adaptive (specialized and derived) immune systems. The splitting of immune functions into two branches occurred with the emergence of jawless vertebrate agnathans (i.e., hagfish) 500 millions of years before present (mybp), and has continued to diversify throughout vertebrate evolutionary radiation (Pancer and Cooper, 2006; Figure 2). The innate immune system is comprised of physical integument barriers, circulating soluble mediators, and an assortment of leukocytes (neutrophils, mast cells, monocytes, macrophages and dendritic cells) and innate lymphoid cells (ILCs) (e.g., natural killer cells), which straddle innate and adaptive immunity to support cross-talk (Eberl et al., 2015; Gasteiger et al., 2017). The innate immune system is versatile and constitutively available, and although it is rapidly inducible by external factors, its function does not depend on precedent exposure. If infections persist, the adaptive immune system mounts a highly discriminating, long-lasting response involving various cell-mediated and humoral lymphocytes, like T cells (e.g., cytotoxic, helper and regulatory), and B cells, which produce immunoglobulins (Igs) (Mashoof and Criscitiello, 2016).

The connections between stress and immune systems are complex, multifaceted, and have deep evolutionary underpinnings that shaped their coordinated and bidirectional communications (Ottaviani, 2011; Figure 2). The immune activation profile is a function of stress exposure duration, wherein protective immuno-enhancements initially occur during acute stress lasting hours to days, but are suppressed over longer periods of chronic stress that last days to months (Martin, 2009). Thus, long-term suppression of immune responses may represent an adaptive down regulation for energy saving to counter allostatic overloads (Martin, 2009). In an ecological scenario where resources are finite, minimizing the activation of calorically expensive immune responses would aid in increasing organismal fitness. However, this could leave the animal at increased risk for infections and diseases.

Neurotransmitters, neuropeptides, and hormones of the SNS and HPA stress axes orchestrate a careful and balanced integration between stress responses and immune activity, and there is a plethora of evidence that the molecular machinery required to respond to these stress-derived molecules are present in immune cells (Sternberg, 2006). Similarly, chemokines, cytokines, and other factors produced by immune cells can regulate the stress response at various points along the HPA axis (Turnbull and Rivier, 1999; Lozovaya and Miller, 2003), including at the hypothalamus (Uehara et al., 1987; Sapolsky et al., 2000), pituitary (Bernton et al., 1987), and adrenal gland (Turnbull and Rivier, 1999) when they are released from resident microglial cells in the brain (Blandino et al., 2009; Hinwood et al., 2012; Kreisel et al., 2014). Cytokines can also interact with the blood brain barrier (BBB) from peripheral sites of inflammation to influence central immune processes by influencing monocyte recruitment to the brain (Banks et al., 1989; Wohleb and Delpech, 2017), or by crossing the BBB when it loses integrity resulting from various disease or inflammatory states (Banks et al., 1989; Gutierrez et al., 1993; Małkiewicz et al., 2019; Rhea and Banks, 2019; Figure 3).

The goal of this bi-directional communication is to co-regulate and coordinate responses to maintain homeostasis. For example, HPA-derived molecules, like CRH, are both immunosuppressive as a hypothalamic regulator of anti-inflammatory GCs (Nesse et al., 2016), but can stimulate macrophages to secrete pro-inflammatory cytokines when released directly from peripheral nerve endings (Agelaki et al., 2002). Pharmacologically antagonizing macrophage CRH receptors (Webster et al., 1996) or chemical sympathectomy of peripheral nerves (Green et al., 1993) also reduces pro-inflammatory cytokine (TNF-α, IL-6 and IL-1) release and reduces inflammation. Interestingly, while these same cytokines stimulate the HPA axis, IL-1 is a particularly potent stimulant of hypothalamic CRH (Berkenbosch et al., 1987; Sapolsky et al., 1987) and ACTH from the pituitary (Bernton et al., 1987). B and T cells also express CRH receptors and produce and secrete the CRH protein (Baker et al., 2003), emphasizing the importance of this critical HPA axis stress molecule as a regulator of immunity.

The ubiquitous expression of GRs on various immune cell types mediates GCs influence on cell phenotype and function (Bellavance and Rivest, 2014), including decreasing maturation of DCs (Matyszak et al., 2000), increasing monocyte anti-inflammatory subtypes (Ehrchen et al., 2007) and macrophage phagocytic capacity (van der Goes et al., 2000), and reducing lymphocyte proliferation and IL-2 levels (Ramírez et al., 1996). These actions thus link the stress-axis to the inflammatory resolution process (Wang et al., 2019). Indeed, GCs are commonly used as acute anti-inflammatory therapeutics to reduce inflammation in humans (Coutinho and Chapman, 2011), domestic mammalian pets (Aharon et al., 2017), and in birds (Gao et al., 2017), further supporting a conserved co-evolution of the two systems.

Although the immunosuppressive and anti-inflammatory actions of GCs cover both acute and chronic exposure scenarios, the beneficial outcomes (i.e., dampening of inflammation) are mostly reserved for short-term scenarios, with detrimental pathologies occurring over the long-term, such as increased infections, prolonged wound-healing, and decreased antibody production following vaccination (Kiecolt-Glaser et al., 1996; Vedhara et al., 2003). Stress-mediated inflammation has also been reported in the skin with increased severity of cutaneous infections (Aberg et al., 2007); in the lungs as asthma from exposure to chronic early-life stressors (Chida et al., 2007); and in various bowel disorders (Buckley et al., 2014). Psychological rodent stressors, like the open field or conditioned aversion stress tests, trigger cytokine release and reduced fevers in the animal (Morrow et al., 1993), while an adrenalectomy (Ruzek et al., 1999; Gómez et al., 2003), hypophysectomy (Edwards et al., 1991) or dosing with GC receptor inhibitors (i.e., RU486) results in elevated fevers (Morrow et al., 1993) and leaves an animal susceptible to infection and sepsis (Sternberg, 2006). Reintroducing GCs following adrenalectomy reverses this susceptibility to infection (Ruzek et al., 1999). Together, this evidence shows that stress-mediated GCs play an important role in maintaining immune homeostatic balance that prevents both over-stimulation or over-suppression of immune responses, particularly during an infection (Sternberg, 2006).

## Ontogeny of the Human HPA Axis

While the HPA axis and immune system exhibit remarkable evolutionary conservation, differences arise when we examine the ontogeny of the HPA axis during gestation across species. For example, the placenta of anthropoid primates, including humans, has evolved the unique ability to produce CRH, forming a cooperative maternal-fetal-placental endocrine unit that is not present in less-derived mammals (Bowman et al., 2001; Power and Schulkin, 2006). Placental CRH is structurally and biologically identical to that of hypothalamic CRH and acts in an allocrine fashion on both maternal and fetal compartments (Karteris et al., 2001; King et al., 2001). However, differences in the expression of placental CRH over the course of gestation exist even between closely related non-human primates, such as monkeys and great apes (Power and Schulkin, 2006), highlighting the divergent influences on HPA axis ontogeny across species. These differences limit our capacity to extrapolate understandings of fetal HPA axis ontogeny from animal models to humans, which remains a key challenge to stress research. As a consequence, this section will focus on the ontogeny of the HPA axis in humans.

The human fetal HPA axis develops progressively over the course of gestation, with key components, including the hypothalamus (Koutcherov et al., 2002), anterior pituitary (Kelberman et al., 2009; Musumeci et al., 2015), and adrenal cortex (Keegan and Hammer, 2002; Xing et al., 2015) being largely differentiated by 8–10 weeks’ gestation (Howland et al., 2017). Formation of the hypothalamic-hypophyseal portal system connecting the hypothalamus and anterior pituitary occurs by 11 weeks (Thliveris and Currie, 1980), with evidence of immunological and biological activity of fetal hypothalamic CRH around 12–13 weeks’ gestation (Ackland et al., 1986). Although, it remains unclear to what degree fetal CRH regulates ACTH production at this stage of development (Howland et al., 2017). In parallel, activity of the adrenal cortex is evident early in gestation (Ishimoto and Jaffe, 2011), but synthesis of cortisol depends on the presence of steroidogenic enzymes, such as 3β-hydroxysteroid dehydrogenase/Δ^4–5^isomerase (3β-HSD) (Goto, 2006). Apart from a transient period of 3β-HSD expression between 7–10 weeks’ gestation, which is thought to be involved in sex differentiation (Goto, 2006), fetal 3β-HSD expression remains low until the end of the second trimester, then rises alongside fetal cortisol production until parturition (Murphy, 1982; Parker et al., 1995; Lockwood et al., 1996). This increase in cortisol is not paralleled by fetal ACTH (Winters et al., 1974; Lockwood et al., 1996), suggesting placental CRH may promote fetal cortisol synthesis either through direct stimulation or by increasing adrenal responsivity to low fetal ACTH levels (Parker et al., 1999; Sirianni et al., 2005; Rehman et al., 2007). Finally, by early in the third trimester, the fetal adrenal cortex represents an elementary version of the adult adrenal cortex (Sucheston and Cannon, 1968; Howland et al., 2017).

**FIGURE 4:**
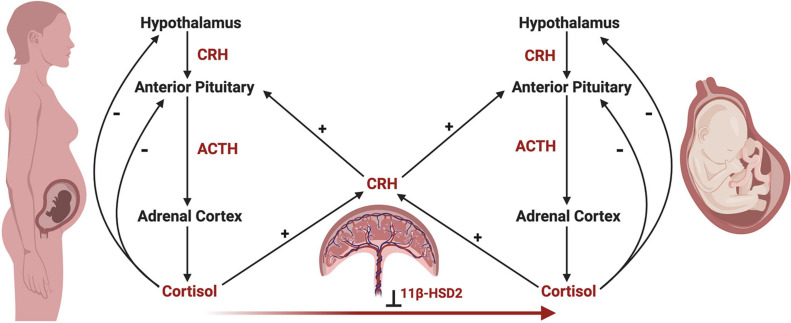
The maternal-fetal-placental endocrine unit. The maternal and fetal HPA axes share a common signal integration site, the placenta, forming the maternal-fetal-placental endocrine unit. This unit represents a complex endocrine network, with the placenta regulating steroidogenic crosstalk between the mother and fetus. In contrast to the negative feedback mechanisms of the maternal and fetal HPA axes, a positive feedback relationship exists between the maternal and fetal HPA axes and placental CRH production. Placental CRH is produced in response to maternal or fetal cortisol and acts on the maternal or fetal anterior pituitary in an allocrine fashion to activate each respective HPA axis. Maternal cortisol and placental CRH aid in regulating the development of the fetal HPA axis through several mechanisms, highlighting the importance of this endocrine network. First, placental CRH is thought to act on the fetal HPA axis via two key mechanisms, by (a) increasing the responsivity of the fetal adrenal cortex to ACTH, and (b) directly stimulating the fetal adrenal cortex to produce cortisol. Second, maternal cortisol may cross the placenta and directly act on the fetal anterior pituitary in an inhibitory fashion, preventing ACTH and cortisol release. However, the influence of maternal cortisol on the fetal compartment is dampened by the actions of 11β-hydroxysteroid dehydrogenase 2 (11β-HSD2), which renders maternal cortisol inactive through oxidation to cortisone. Under the influence of maternal stress, the regulatory actions of the maternal-fetal-placental unit may break down, causing shifts in hormone levels such as increased placental CRH or decreased 11β-HSD2 resulting in higher levels of cortisol in the fetal compartment via fetal production or transfer of maternal cortisol across the placenta, respectively. This may have important implications on fetal HPA axis ontogeny and increase the risk of disease development later in life.

Development of the fetal HPA axis relies on maternal and placental signals via the maternal-fetal-placental unit, which represents a complex endocrine network, with the placenta acting as an integration site for signals between the mother and fetus (Reis and Petraglia, 2001; Howland et al., 2017). Unlike the negative feedback actions of cortisol on maternal and fetal HPA axes, cortisol of maternal or fetal origin increases placental CRH production, which in turn increases maternal and fetal HPA axis activity in a positive feedback manner (Robinson et al., 1988; Wadhwa et al., 1997; Mastorakos and Ilias, 2003; Figure 4). In parallel, the placenta functions to reduce the amount of maternal cortisol that reaches the fetus through enzymatic oxidation of cortisol to cortisone by 11β-hydroxysteroid dehydrogenase type 2 (11β-HSD2), rendering it inactive (Beitins et al., 1973; Benediktsson et al., 1997; Schoof et al., 2001).

Placental 11β-HSD2 expression fluctuates over gestation and differs with fetal sex, with low levels during early and late gestation, and in pregnancies with females (Carpenter et al., 2017), enabling increased transfer of maternal cortisol to the fetus (Beitins et al., 1973; Benediktsson et al., 1997; Schoof et al., 2001; Murphy and Clifton, 2003). During early gestation, maternal cortisol is thought to suppress fetal ACTH production (Pepe and Albrecht, 1995), but excessive cortisol exposure during this period may negatively impact the developing fetus (Davis and Sandman, 2010; Buss et al., 2012). As placental 11β-HSD2 expression rises mid-gestation, the fetal pituitary is disinhibited, enabling ACTH production (Pepe and Albrecht, 1995; Schoof et al., 2001), which then stimulates adrenocortical development (Ishimoto and Jaffe, 2011). During late gestation, placental 11β-HSD2 levels decrease and maternal cortisol increases, as cortisol plays a key role in facilitating maturation of fetal organ systems prior to birth (Ishimoto and Jaffe, 2011; Howland et al., 2017). However, in the face of maternal stress, placental 11β-HSD2 levels may further decline, allowing increased amounts of maternal cortisol to reach the fetus and potentially have detrimental effects, particularly in females (Giesbrecht et al., 2013; Carpenter et al., 2017). Females have also been shown to have higher cord blood cortisol levels at birth, indicating higher circulating cortisol in the fetal compartment. However, this sex difference was shown to be independent of fetal production, and rather, of maternal or placental origin (Giesbrecht et al., 2016). Together, the placenta plays a vital role in the integration and regulation of both the maternal and fetal stress axes as the interface between these compartments, including being implicated in sex differences in fetal HPA axis development and steroid exposure. High levels of physiological or psychological maternal stress during the prenatal period has the potential to significantly affect fetal HPA axis development and programming through three primary actions on the maternal, placental, and fetal compartments (Howland et al., 2017). First, maternal stress increases maternal cortisol levels (Giesbrecht et al., 2012, 2013), which may enter fetal circulation and alter the developmental programming of the fetal HPA axis, especially early in pregnancy (Davis and Sandman, 2010) and in females (Carpenter et al., 2017). Second, elevated maternal cortisol can act indirectly on the fetus by increasing placental CRH (Sandman et al., 2006), which may then stimulate the fetal adrenal glands to increase cortisol production or alter adrenal sensitivity to ACTH (Lockwood et al., 1996; Sirianni et al., 2005; Rehman et al., 2007). Elevated placental CRH may also cross the fetal BBB (Kastin and Akerstrom, 2002) and act on CRH receptors in developing brain regions involved in regulating the HPA axis (Koutmani et al., 2013; Howland et al., 2017). Third, maternal stress may lead to downregulation of placental 11β-HSD2, enabling greater amounts of maternal cortisol to reach the fetus (Benediktsson et al., 1997; Howland et al., 2017). Similar to upregulated placental CRH, elevated levels of maternal cortisol circulating in the fetal compartment may alter the programming of the HPA axis in a sex-dependent manner (Cottrell and Seckl, 2009; Giesbrecht et al., 2016; Carpenter et al., 2017), primarily by altering the abundance or sensitivity of GRs (Welberg et al., 2001; Noorlander et al., 2006). Sex-dependent shifts in the activity, balance, and programming of GRs along the HPA axis during fetal development may result in postnatal dysregulation of key physiological systems (Giesbrecht et al., 2016; Rash et al., 2016), laying the foundation for sex differences in disease development later in life (de Kloet et al., 1998; Reynolds, 2013).

Beyond the prenatal period, the development of the HPA axis continues into early life, with stressors and environmental exposures continuing to influence its maturational trajectory either positively or negatively (Lai and Huang, 2011; Mooney-Leber and Brummelte, 2017; Sominsky et al., 2018; Agorastos et al., 2019). The effects of these exposures on the developmental programming of the HPA axis may vary depending on timing, duration, and severity, with a multitude of research highlighting these relationships that will not be examined in this paper (Kuhlman et al., 2017; Agorastos et al., 2019). Recently, the gut microbiome has been recognized as a critical environmental exposure that influences mammalian developmental trajectories, with global influences on the maturation of major body systems, including stress axes (Foster et al., 2017). Subsequently, we will focus on the bidirectional relationship between the developmental trajectories of the gut microbiota and the HPA axis, recognizing the early life microbiota as an exposure that influences stress axis programming, while the establishment of the microbiota itself is influenced by stress. We will then examine the consequences of perturbations to these systems on each other and on disease development.

## The Early-Life Microbiome

The gut microbiome represents a complex ecosystem with significant functional capacity, as these microorganisms encode a significant amount of genetic material beyond that of the human genome (Lozupone et al., 2012). This genetic diversity gives the microbiome substantial adaptive capacity, evidenced by its ability to persist with changes in environmental exposures (Lozupone et al., 2012; Armour et al., 2019). The gut microbiome and the host exist in a mutually beneficial relationship, in which the microbiome performs functions essential to human health in exchange for a nutrient-plentiful environment to reside (Flint et al., 2012). This includes, but is not limited to, extensive metabolic activities (Oliphant and Allen-Vercoe, 2019), directing immune system development and responses (Martin et al., 2010; Belkaid and Hand, 2014), maintenance of gastrointestinal homeostasis and integrity (Forgie et al., 2019; Lobionda et al., 2019), and competitive exclusion of pathogens (Pickard et al., 2017).

The composition of the gut microbiota in early life is primarily determined by the ecological frameworks of selection and priority effects. Along the length of the gastrointestinal tract, environmental factors such as pH, motility, temperature, and nutrient availability select for the consortium of microbes that occupy a given ecological niche, with the majority of microbes residing within the colon (Hillman et al., 2017; Chang and Kao, 2019). In parallel, the order and timing of initial colonizers helps direct the formation of niches via metabolic activities and the generation of cross-feeding networks, and through these processes, may influence the success of subsequent colonizers and the course of ecosystem development, known as priority effects (Fukami, 2015; Sprockett et al., 2018). However, defining microbial community compositions as health- or disease-promoting is challenging, as functional redundancy - the ability of one microorganism to perform analogous functions to another - across microbial taxa limits our understanding of optimal community composition (Human Microbiome Project Consortium, 2012; Louca et al., 2018). This is emphasized by the degree of inter-individual variability in microbiota composition despite functional similarities, as each individual accommodates a unique community of approximately 160 species (Flint et al., 2012; Human Microbiome Project Consortium, 2012; Marchesi et al., 2016). An emerging link exists between early-life gut microbial colonization patterns and the development of physiological systems across the body (Stiemsma and Michels, 2018), suggesting ecological perturbations to the microbiota during this time have the potential to alter host developmental programming and lifelong health trajectories.

In humans, initial colonization of the infant gastrointestinal tract begins following rupture of the amniotic sac, as the placenta is a sterile environment, with the exception of uncommon pre-labor pathogens (de Goffau et al., 2019). During parturition, infants are first exposed to and rapidly colonized by a variety of microbes, with distinct colonization patterns existing between those born vaginally or by C-section. Vaginally delivered infants are colonized by microbes residing within the vaginal canal and maternal feces, whereas infants delivered via C-section are colonized by those associated with the skin (Palmer et al., 2007; Dominguez-Bello et al., 2010; Reyman et al., 2019). The gut microbiota then undergoes rapid expansion and periods of transition until about 2–3 years of age, as colonizing microbes shape the intestinal environment to be suitable for a diverse community of anaerobic species in a process of ecological succession (Koenig et al., 2011; Laforest-Lapointe and Arrieta, 2017). At this point, the microbiota stabilizes into a mature ecosystem and remains largely compositionally consistent throughout adulthood (Stewart et al., 2018; Xu et al., 2019).

Colonization patterns during these first few years are influenced by a number of prenatal and postnatal factors, including maternal health (Chu et al., 2016; Wang J. et al., 2018), gestational age (La Rosa et al., 2014; Korpela et al., 2018), delivery mode (Palmer et al., 2007; Dominguez-Bello et al., 2010; Reyman et al., 2019), nutritional source (Timmerman et al., 2017; Borewicz et al., 2019), antibiotic exposure (Yassour et al., 2016), geography (Gupta et al., 2017), and both prenatal (Zijlmans et al., 2015; Hantsoo et al., 2019; Hechler et al., 2019; Naudé et al., 2020) and early-life stress (Browne et al., 2019; D’Agata et al., 2019). This early period of microbiota development is regarded as a critical window, during which perturbations to the ecological patterns of colonization have the potential to disrupt microbial signals involved in directing normal developmental programming, including that of neural, immune, and metabolic host physiology (Borre et al., 2014; O’Mahony et al., 2017; Stiemsma and Michels, 2018). The ecological frameworks of selection and priority effects become increasingly relevant in light of this critical window. In the absence of microbial signals characteristic of a typically developing microbiota, shifts in developmental programming may occur, with long-lasting implications for human health and disease (O’Mahony et al., 2017; Stiemsma and Michels, 2018).

Substantial research efforts have sought to define the patterns of microbial colonization occurring in the first years of life (Wopereis et al., 2014; Wampach et al., 2017; Derrien et al., 2019; Fouhy et al., 2019), in an attempt to understand whether these patterns are conserved, and if so, be able to discern between typical and abnormal ecosystem succession. However, these processes are only partly understood, underpinned by the substantial number of exposures influencing colonization patterns and inter-individual variability in microbiota composition. One exposure that has only recently been recognized as critical to microbial colonization is stress, including both prenatal and postnatal exposures. The relationship between the gut microbiome and stress is bidirectional, with the patterns of microbial colonization in early life being involved in shaping stress axes, and stress exposures being sufficient to induce shifts in microbial ecosystems (Foster et al., 2017). These microbial alterations may diverge from that of conserved colonization patterns, and thus, lead to alterations in microbial signals essential to normal developmental programming.

Maternal stress can have broad effects on both fetal and infant development through its influence on the fetal HPA axis (Howland et al., 2017) and maternal vaginal (Culhane et al., 2001), fecal (Zijlmans et al., 2015; Hantsoo et al., 2019; Hechler et al., 2019; Naudé et al., 2020) and milk microbiotas (Browne et al., 2019). In the vaginal canal, stress shifts the microbiota away from *Lactobacillus* dominance (Culhane et al., 2001; Amabebe and Anumba, 2018), heightening the risk of poor pregnancy outcomes and preterm birth (Dingens et al., 2016; Shimaoka et al., 2019). In parallel, alterations to the maternal fecal microbiota have been reported (Hantsoo et al., 2019; Hechler et al., 2019; Naudé et al., 2020), although inconsistent taxonomic shifts have been observed across studies. For example, prenatal anxiety has been associated with increased *Oxalobacter*, *Rothia*, and Gram-positive cocci, such as *Staphylococcus* (Hechler et al., 2019), whereas maternal cortisol responses have been positively correlated with *Rikenellaceae* and *Dialister* abundance, and negatively correlated with *Bacteroides* (Hantsoo et al., 2019). Interestingly, shifts in *Rikenellaceae* often emerge in animal models of stress (Pusceddu et al., 2015; Gur et al., 2017; Tsilimigras et al., 2018). Further, maternal stress has been linked to significantly reduced diversity and the absence of typical compositional shifts in the milk microbiota over lactation (Browne et al., 2019), with further differences being observed between mothers who deliver vaginally versus by C-section (Cabrera-Rubio et al., 2012). This is thought to be related to altered hormonal signals and experiences of physiological stress as consequence of delivery mode (Cabrera-Rubio et al., 2012). Together, shifts in the maternal vaginal, fecal, and milk microbiota are highly relevant to vaginally born and/or breastfed infants as sources of initial microbial colonizers, with alterations in these pioneer species potentially having far-reaching consequences on the developmental trajectory of the microbiota due to priority effects (Fukami, 2015). In fact, infants of prenatally stressed mothers have been shown to have reductions in beneficial lactic acid bacteria and *Bifidobacteria*, and increases in Proteobacteria (Zijlmans et al., 2015; Hantsoo et al., 2019), highlighting the importance of this relationship.

Clinical evidence of the bidirectional relationship between stress and the gut microbiome has only begun to emerge (Zijlmans et al., 2015; Browne et al., 2019; D’Agata et al., 2019; Hantsoo et al., 2019; Hechler et al., 2019; Naudé et al., 2020), but it is apparent that the interplay between alterations in stress axis activity and microbial colonization patterns have the potential to significantly influence developmental programming in early-life at both the maternal and infant levels. This complex relationship is mediated by gut-brain communication mechanisms, including both microbial- and host-derived signals (Clarke et al., 2014; Cryan et al., 2019), which alert microbial ecosystems and the host of the need to adapt accordingly in the presence of different environmental exposures. Research examining this relationship in clinical cohorts is needed to further elucidate how stress may alter conserved colonization patterns, and likewise, how shifts in microbial colonization as a result of other early-life exposures, such as antibiotics, may inform the development of stress axes, and the mechanisms of gut-brain signaling involved in these adaptations.

## Microbial Communication Mechanisms Within the Gut-Brain Axis

Microbial mechanisms that mediate communication between the gut microbiome and the brain are varied and not fully understood, but involve various chemical messengers such as cytokines, chemokines, catecholamines, short chain fatty acids (SCFA), and other neuroactive molecules (e.g., serotonin, melatonin, GABA) derived by commensal gut microorganisms and the host (Sudo, 2012). Multiple direct and indirect neural, humoral, and hormonal pathways have also been identified, creating an intricate signaling network that exerts its effects not only on the gut, but also on the immune and stress response systems (Figure 3). There have been a recent plethora of excellent and comprehensive reviews (Sudo, 2014; de Weerth, 2017; Foster et al., 2017; Farzi et al., 2018; Sylvia and Demas, 2018; Wang S. et al., 2018; Cryan et al., 2019) that explore this important topic in depth. Thus, for this section, the most significant contributors will be highlighted to provide context and breadth of their roles in gut-brain communication, stress responses, and early-life development.

### Neurotransmitters, Neuromodulators, and the Nervous System

Pioneering studies in the 1990s showed that microbes could not only respond to vertebrate hormones and neuromodulators (Lyte, 1993), but were later shown to also synthesize potent neuromodulators and neurotransmitters (e.g., GABA, serotonin, dopamine, and acetylcholine) (Lyte, 2014) commonly produced by mammals, indicating bi-directional communication with the host gut tissue using molecules that are associated with modulating mood, behavior, and cognition (Cryan et al., 2019).

Serotonin (5-HT), for example, is a critical biogenic indoleamine that is synthesized from the amino acid, tryptophan. It functions as a neurotransmitter in the brain and is well-documented to regulate mood and cognition (McLean et al., 2007). In the periphery, 5-HT is heavily involved in the regulation of intestinal motility and secretion (Foster et al., 2017). In fact, 95% of 5-HT is produced by enterochromaffin cells in the gut mucosa, with recent evidence showing that the gut microbiome can regulate 5-HT synthesis (Yano et al., 2015). Monoamine metabolism is also identical in all vertebrates (Winberg and Nilsson, 1993), suggesting an ancient host-microbe communication mechanism that utilizes key mood-regulating monoamine neurotransmitters.

Gut microbes also communicate with the brain by directly utilizing vagus nerve signaling to send messages to the CNS (Fülling et al., 2019). Approximately 90% of the vagus nerve at the level of the gut consists of afferent fibers that relay information from the viscera to the brain (Powley et al., 2019), providing a key bi-directional conduit for microbial signals to influence brain activity and behavior. It has been demonstrated that rat vagal afferent neurons in the gut express GABA receptors (Ashworth-Preece et al., 1997) that are likely targets for GABA produced by commensal *Lactobacillus* and *Bifidobacterium* (Barrett et al., 2012). Further, long-term supplementation with *Lactobacillus rhamnosus* reduced circulating GCs and stress-related anxiety disorders in mice by inducing widespread changes in GABA receptor mRNA expression throughout the vagus nerve and in various regions of the brain (Bravo et al., 2011), with vagotomy abolishing these effects. *Ex vivo* electrophysiological studies also show that direct intraluminal infusion of *L. rhamnosus* into the small intestine of mice increases vagal afferent nerve bundle firing within minutes, substantiating a potential psychoactive role for gut microbes (Perez-Burgos et al., 2012). Early-life microbiome colonization also affects vagus nerve functional responses (Forsythe et al., 2014; Wang S. et al., 2018), as well as the developmental trajectory of brain regions in mice involved in motor control, anxiety behaviors, and cognition by regulating synaptogenesis (Diaz Heijtz et al., 2011) and myelination (Hoban et al., 2016).

### Immune Factors

Vagal afferent gut terminals exist in close contact with the mucosal immune system of the intestine, known as the gut-associated lymphoid tissue (GALT) (Patterson et al., 2002), where GALT immune cells act as mediators between microbes in the gut lumen and the CNS for important bi-directional communications between the two systems to regulate neuroendocrine activity and behaviors (de Weerth, 2017). It is suggested that the GALT can recognize and communicate with the microbiota (Mayer, 2011), as the GALT contains 70–80% of the body’s immune cells and is largely tolerant to commensal gut microbes, but develops effector functions toward pathogenic ones. The GALT secretes pro-inflammatory cytokines when pathogenic microbial antigens like lipopolysaccharide (LPS) from pathobionts penetrate beyond the protective epithelial layers, and ultimately, activate the HPA axis by binding to toll-like receptors (TLR) (Navarra et al., 1991; Schmidt et al., 2003; Ratnayake et al., 2013). Mice lacking a microbiota have abrogated TLR4 developmental expression in the gut (Hörmann et al., 2014; Inoue et al., 2017), while mice deficient for TLR4 or myeloid differentiation factor 88 (MyD88) (critical for signal transduction cascades) have an abrogated HPA axis response to Gram-negative bacteria (Gosselin and Rivest, 2008). Neonatal rats exposed to *Salmonella enteritidis* between postnatal days 3 to 5 also have significantly altered neuroendocrine stress responses along the whole HPA axis as adults (Shanks et al., 1995), including elevated ACTH responses to restraint stress, decreased negative feedback sensitivity to GCs, elevated resting CRH levels and mRNA in the hypothalamic PVN, and reduced GR density in several brain regions.

Apart from LPS, other microbiota-derived constituents, like cell wall peptidoglycans, can translocate beyond the BBB during postnatal development in healthy mice and activate neuronal pattern-recognition receptors (PRRs), like nucleotide-binding oligomerization domain-containing protein 2 (NOD2) and peptidoglycan recognition protein 2 (pglyrp2), both of which are involved in neurodevelopmental processes (Arentsen et al., 2017). NOD2 is highly expressed by microglia and astrocytes, and these specialized neuroimmune cells are not only critical for inflammatory responses to pathogens (Chauhan et al., 2009), but also for neuronal tissue development during early life (Schafer and Stevens, 2013). These novel findings constitute another signaling mechanism by which commensal gut microbes mediate communication with the brain to influence neuroendocrine development and behavior.

### Microbial By-Products

Gut bacteria convert host-indigestible dietary fibers (i.e., prebiotics) into SCFAs (e.g., acetate, propionate and butyrate) through fermentation in the gut. These microbial-derived metabolites are essential for host health and are utilized for many physiological functions, including epigenetic changes in chromatin structure (Licciardi et al., 2011), gut functioning (Parada Venegas et al., 2019), host metabolism (Koh et al., 2016), blood pressure (Miyamoto et al., 2016), neuroimmune function (Erny et al., 2015), circadian rhythms (Tahara et al., 2018), and are implicated in stress and behavior.

Findings from van de Wouw et al. (2018) showed that SCFA supplementation in mice alleviated long-term anhedonia (depression-like state), diminished HPA hyper-responsiveness, and improved gut barrier function by reducing permeability. In a separate study, butyrate supplementation restored brain function in cognitively impaired mice resulting from a high-fat diet by recovering cerebral functional connectivity, cerebral blood flow, neuroinflammation and reduced the number of active microglia in the brain to control levels (Arnoldussen et al., 2017). A recent cohort study in humans reported butyrate-producing *Faecalibacterium* and *Coprococcus* bacteria were consistently associated with higher quality of life indicators, and were depleted in those with depression, even after correcting for antidepressants (Valles-Colomer et al., 2019). Interestingly, cerebrospinal fluid is implicated as a possible transport route for SCFAs like acetate to directly access and target the brain (Nagashima et al., 2010; Perry et al., 2016).

More recently, gut fungi have been implicated as important colonizing microbes for appropriate host immune development and functionality in mice by regulating the colonization status of gut bacteria, and in managing peripheral inflammatory diseases like asthma and colitis (van Tilburg Bernardes et al., 2020). Commensal fungal species, like *Candida albicans*, were also shown to perturb the HPA axis in rats by manipulating the endocannabinoid system, which regulates the stress axis and anxiety-like behaviors. Rats colonized with *C. albicans* had increased basal circulating corticosterone and anxiety, relative to controls (Markey et al., 2020). Behavioral implications of directly communicating with the brain from the gut are significant and intriguing, but require focused studies to determine the mechanistic modalities from these findings.

### Microglial Cells

Recent evidence has identified microglial cells as key regulators of stress activation and inflammation in the brain (Lenz and Nelson, 2018). Microglia are by far the most abundant and important immune cells in the CNS. They function as scavenging tissue macrophages to maintain tissue homeostasis, and are involved in the propagation of brain inflammatory responses by releasing cytokines and chemokines that not only recruit local immune cells, but also monocytes from the periphery, to help fight infections and clear cell debris (Lenz and Nelson, 2018). Microglia are receptive to many peripheral and gut-derived chemical messengers such as cytokines, chemokines, catecholamines and other neuroactive molecules (e.g., serotonin, melatonin, GABA), and are important for proper early-life brain development and remodeling by pruning synapses (Schafer and Stevens, 2013; Erny et al., 2015). However, over-active microglia lead to stress-related anxiety and depressive disorders (Ramirez et al., 2015; Wohleb and Delpech, 2017; Weber et al., 2019), as well as cognitive deficits and neural inflammation following septic infection (Andonegui et al., 2018). Chemically blocking or eliminating stress-experienced microglia prevents both immune reactivity in the brain and anxiety recurrence following a stress challenge (Weber et al., 2019), suggesting that microglia may directly regulate stress-related behaviors.

Interestingly, microglia from germ-free (GF) mice have altered phenotypic proportions with more cells resembling the ramified, surveying phenotype, and had globally defective immune responses to infection challenges (Erny et al., 2015). These altered phenotypic proportions were rescued with SCFAs supplementation (Erny et al., 2015), insinuating that gut microbial by-products, such as SCFAs are critical for the proper proportionality of microglial cell morphologies. Moreover, transgenic mice deficient for the SCFA receptor, free fatty acid receptor (FFAR2), also had defective microglia that mirrored those observed in GF mice (Erny et al., 2015), despite microglia not expressing FFAR2. Therefore, SCFAs may indirectly modify microglia phenotype and function via their actions on FFAR2 expressed in other tissues and cells. Recently, Luck et al. (2020) showed that colonizing neonatal GF mice with a consortium of *Bifidobacterium* was critical to the normal phenotypic development of microglial cells, whereas those not colonized had abundant phagocytizing ameboid phenotypes that persisted into adulthood. Further, uncolonized GF mice had disrupted functional neuronal circuits, increased synaptic densities, and decreased firing rates. Since *Bifidobacterium* produce large quantities of the SCFA, acetate, this may be a mechanism by which microbes mediate early-life development of microglia (Luck et al., 2020). Similarly, prebiotic fiber treatment with 10% oligofructose attenuated impaired hypothalamic microglial activation in neonatal mice exposed to early-life antibiotics (Cho et al., 2020). Despite this recent evidence, the precise microbial pathways involved, and which microbial species or features affect homeostatic regulation of neuronal development and stress and immune systems, remains unclear and a significant research gap to explore.

## Microbiome Alterations and Stress-Axis Activity

Early-life environmental exposures play a critical role in the development and functional programming of central neural circuitry, including the HPA axis (Foster et al., 2017), with sex and inter-individual differences in these exposures linked to the degree of stress axis activity and vulnerability to developing stress-related disorders later in life (Cottrell and Seckl, 2009; Shields and Slavich, 2017). Environmental exposures have primarily been considered external in nature, such as the stressful life experiences of abuse, poverty, neglect, and familial conflict, collectively referred to as adverse childhood experiences (ACEs) (Danese and McEwen, 2012). However, novel understandings generated in the gut-brain axis literature suggest, through its communication mechanisms with the brain, that the gut microbiome constitutes another environmental exposure (Cryan et al., 2019). The bi-directionality of this gut-brain relationship is highlighted by evidence that stress exposure shifts the structural and functional status of the microbiome, while in parallel, the absence of early-life microbial exposures (GF status) alters stress axis activity and the developmental programming of stress circuitry (Sudo et al., 2004; Heijtz et al., 2011; Crumeyrolle-Arias et al., 2014; Gur et al., 2017; Huo et al., 2017; Jašarević et al., 2018; Cowan et al., 2019; Lyte et al., 2020). Through this lens, a complex interplay between early-life exposures to stress and microbial colonization is emerging as a central component in the developmental programming and life-long activity of the neuroendocrine stress response.

Evidence of the gut microbiome being critically involved in the regulation of stress physiology early in life has primarily emerged from animal models (Table 1). In particular, GF animals enable improved understandings of the role microbes play in development and allow for manipulation of colonization status at defined developmental timepoints (Luczynski et al., 2016). Seminal work by Sudo et al. (2004) revealed, following a 1 h restraint stress, that male GF mice displayed significantly higher HPA axis activity, as measured by elevated plasma ACTH and corticosterone (equivalent to cortisol in humans) relative to specific pathogen free (SPF) mice and those mono-associated with the beneficial microbe, *Bifidobacterium infantis*. Interestingly, this exaggerated response was partially rescued following colonization of GF animals only at an early developmental time point. The absence of a rescue effect with microbial colonization at later stages implied a critical window exists during which the microbiome is able to influence the developmental programming of the HPA axis. Subsequent studies in GF animals have revealed similar findings, including elevated CRH mRNA in the hypothalamus and reduced GR mRNA expression in the hippocampus, indicative of greater HPA axis activity and reduced potential for negative feedback inhibition (Sudo et al., 2004; Crumeyrolle-Arias et al., 2014; De Palma et al., 2015; Huo et al., 2017). While a major limitation in the field is the abundance of research performed exclusively in male animals, reports of sex-dependent effects are beginning to emerge, with females being more vulnerable to prolonged HPA axis alterations following acute stress exposure (Lyte et al., 2020).

**TABLE 1:** Studies examining early-life stress and the microbiota-gut-brain axis, categorized by model organism.

**Authors**	**Strain/Sex**	**Stress Paradigm**	**Intervention**	**Effect**
***Mice***				
Bailey et al., 2011	CD-1 ♂	Prolonged RS for 12 h per night for 7 consecutive nights	*Citrobacter rodentium* infection	↓ alpha diversity and ↑ colitis in RS mice ↑ fecal shedding of *C. rodentium* in first 3 weeks of infection
Bharwani et al., 2016	C57BL/6 ♂	Chronic social defeat stress for 10 days		↓ alpha diversity, *Akkermansia*, and *Coriobacteriaceae* Complex shifts in Firmicutes:Bacteriodetes ↑ serum IL-6 5 days following SDS ↓ fatty acid, tyrosine, and tryptophan metabolism and biosynthesis
De Palma et al., 2015	C57BL/6 ♂♀	MS from PND4-21 for 3 h per day		↑ serum corticosterone in MS GF mice relative to GF controls, no sex effect ↓ hippocampal BDNF in MS GF mice relative to GF controls Shifts in microbiome in SPF MS mice maintained into adulthood ↓ *Mucispirillum* and ↑ *Lachnospiraceae* in MS SPF mice relative to SPF controls Shifts in glutamate, tryptophan, tyrosine, and fatty acid metabolism in MS mice
Gur et al., 2017	C57BL/6 ♂♀	RS from E10-16 for 2 h per day		Shifts in microbial relative abundance in RS dams Shift in Firmicutes:Bacteroidetes in PNS offspring, with ↓ *Bifidobacteriaceae*, *Rickenellaceae*, and *S24-7* in females ↓ amygdalar BDNF and ↑ anxiety-like behavior in PNS adult females
Gur et al., 2019	C57BL/6 ♂	RS from E10-16 for 2 h per day		↓ serotonergic metabolism and ↑ CRH in cortex of PNS adult offspring Shifts in microbial relative abundance in PNS adult offspring with ↓ *Bacteroides* and *Parabacteroides*
Huo et al., 2017	Kunming ♂	Chronic RS for 4 h per day for 21 days		↑ CRH, ACTH and corticosterone in GF RS mice relative to SPF RS mice ↓ MR and GR in GF RS mice relative to GF control
Jašarević et al., 2015	C57BL/6:129 ♂♀	Chronic variable stress from E1-7		Positive correlation between ↓ vaginal *Lactobacillus* in PNS dams and offspring ↑ *Bacteroides* and *Clostridium* and ↓ *Lactobacillus* in PNS male offspring resembled control females; PNS females resembled control males ↓ PVN amino acids in PNS males
Jašarević et al., 2017	C57BL/6:129 ♂♀	Chronic variable stress from E1-7		↑ *Rikenellaceae* and *Odoribacter* and ↓ *Bacteroides* early in gestation in vaginal microbiome, ↑ *Desulfovibrionaceae* and *Mucispirillum* late in gestation ↓ vaginal and fecal *Lactobacillus* at PND2 in PNS dams and offspring, respectively Microbiome of PNS males significantly different than females and control males ↑ *Odoribacter*, *Desulfovibrio*, *Flexispira*, and *Mucispirillum* in PNS offspring ↑ *Lachnospiraceae* and *Clostridiales* in PNS males
Jašarević et al., 2018	C57BL/6:129 ♂♀	Chronic variable stress from E1-7		↑ plasma corticosterone in PNS male offspring, no effect in females ↓ *Lactobacillus* in PNS offspring associated with altered free amino acid in PND2 hypothalamus ↑ *Escherichia coli*, *Streptococcus acidominimus*, *Peptococcaceae*, *Streptococcus thoraltensis*, and *Staphylococcus lentus* in PNS offspring Maternal vaginal microbiome unable to rescue the effects of PNS
Lyte et al., 2020	C57BL/6 ♂♀	Acute RS for 15 min		↑ plasma corticosterone in RS GF mice with prolonged ↑ in females relative to RS exGF and CON ↑ colonic 5-HT in RS exGF and CON males ↓ frontal cortex 5-HT in RS CON males
Sudo et al., 2004	BALB/c ♂	Acute RS for 1 h		↑ plasma ACTH and corticosterone in RS GF mice relative to RS SPF mice, ameliorated by monoassociation with *Bifidobacterium infantis* or recolonization of GF mice only at an early developmental timepoint ↓ BDNF in cortex and hippocampus of RS GF mice relative to RS SPF mice
Tsilimigras et al., 2018	CF-1 ♂♀	Daily rotation between RS for 30 min and forced swim test for 19 days		↑ plasma corticosterone in stressed females Shifts in microbial relative abundance with stress and sex ↑ *Ruminococcus gnavus, Odoribacter and Lachnospiraceae in stressed females*
van de Wouw et al., 2018	C57BL/6 ♂	Social defeat and intermittent overcrowding psychosocial stress for 3 weeks	SCFA (sodium acetate, sodium propionate, and sodium butyrate)	↑ CRH, plasma corticosterone, and MR expression in stressed mice, dampened by SCFA supplementation ↑ intestinal permeability in stressed mice, ameliorated by SCFA supplementation ↓ *Ruminococcaceae and* ↑ *Prevotellaceae in stressed mice*
***Rats***				
Ait-Belgnaoui et al., 2012	Wistar ♀	Partial RS for 2 h	*Lactobacillus farciminis*	↑ hypothalamic CRH mRNA, serum ACTH, corticosterone, and intestinal permeability in stressed rats prevented by *L. farciminis* treatment ↑ hypothalamic IL-1β, Il-6, and TNF-α mRNA expression in stressed rats prevented by *L. farciminis* treatment
Cowan et al., 2019	SD ♂	MS from PND2-14 for 3 h per day	*Lactobacillus rhamnosus* R0011 and *Lactobacillus helveticus* R0052	MS rats display mature mPFC engagement during fear expression and inhibition, prevented by probiotic treatment
El Aidy et al., 2017	5-HTT^+/+^, 5-HTT^±^, and 5-HTT^–/–^ Wistar ♂♀	MS from PND2-15 for 6 h per day		Shift in Firmicutes:Bacteroidetes in MS rats Shift toward inflammatory microbial community with ↑ *Desulfovibrio*, *Mucispirillum*, and *Fusobacterium* in MS 5-HTT^–/–^ rats
Geng et al., 2020	SD ♀	Communication box stress for 28 days		↑ serum ACTH and NE in cortex, amygdala, and hippocampus of stressed rats Shift in Firmicutes:Bacteroidetes in stressed rats ↑ *Prevotellaceae, Odoribacter, Desulfovibrio, and Phascolarctobacterium and*↓ *Ruminococcaceae in stressed rats*
Golubeva et al., 2015	SD ♂	RS from E14-20 for 45 min three times per day		↑ and prolonged plasma corticosterone in PNS offspring ↓ *Lactobacillus* and ↑ *Oscillobacter*, *Anaerotruncus*, and *Peptococcus* in PNS offspring
Moussaoui et al., 2017	Wistar ♂♀	LNS from PND2-10; MS from PND2-9 for 15 min per day		↑ corticosterone and intestinal permeability in LNS females ↓ alpha diversity and fiber-degrading, butyrate-producing, mucus-resident microbes and ↑ G + cocci Plasma corticosterone negatively correlated with *Akkermansia* in LNS rats
Neufeld et al., 2019	SD ♂	MS from PND2-12 for 3 h per day; acute RS at 13.5 weeks for 30 min	PDX and GOS and/or *Lactobacillus rhamnosus* GG (LGG)	↑ anxiety-like behavior and ↓ hippocampal-dependent learning in MS rats, attenuated by enriched diet ↓ hippocampal MR and GR with prebiotic and/or LGG in non-stressed rats and ↓ MR and GR with LGG in MS rats
Provensi et al., 2019	Wistar ♂	RS from PND30-45 for 1 h per day then housed with new partner until PND45	ω-3 PUFA (EPA/DHA/DPA) and vitamin A enriched diet	↓ *Lachnospiraceae* and *Ruminococcaceae* and ↑ *Eubacterium* and *Coriobacteriaceae* in stressed rats, prevented by enriched diet ↑ alpha diversity in stressed rats fed enriched diet ↓ SCFA (butyrate, valerate, isobutyrate) in stressed rats ↓ hippocampal BDNF in stressed rats, prevented by enriched diet
Pusceddu et al., 2015	SD ♀	MS from PND2-12 for 3 h per day	Low or high dose ω-3 PUFA (EPA/DHA)	↑ *Akkermansia*, *Flexibacter* and *Prevotella* and ↓ *Rickenella* in MS rats Plasma corticosterone positively correlated with *Rickenella* and negatively with *Akkermansia* in MS rats Shift in Bacteroidetes: Firmicutes in MS rats, reversed by PUFA ↑ *Butyrivibrio* and Actinobacteria and ↓ Proteobacteria with high dose PUFA in MS rats
***Monkeys***				
Bailey et al., 2004	*Rhesus (Macaca mulatta)* ♂♀	Acoustic startle 5 times per week in early (E50-92) or late (E105-147) gestation		↑ plasma cortisol in PNS dams ↓ *Lactobacillus* in early and late PNS offspring for first 6 months of life ↓ *Bifidobacteria* in late PNS offspring for first 6 months of life

*Restraint stress, RS; embryonic, E; social defeat stress, SDS; maternal separation, MS; postnatal day, PND; germ free, GF; specific pathogen free, SPF; brain derived neurotrophic factor, BDNF; prenatally stressed, PNS; corticotropin releasing hormone, CRH; adrenocorticotropin hormone, ACTH; mineralocorticoid receptor, MR; glucocorticoid receptor, GR; paraventricular nucleus, PVN; conventionally colonized, CON; short chain fatty acids, SCFA; Sprague Dawley, SD; medial prefrontal cortex, mPFC; norepinephrine, NE; limited nesting stress, LNS; Gram positive, G+; polydextrose, PDX; galacto-oligosaccharides, GOS; polyunsaturated fatty acids, PUFA.*

Hypothalamus-pituitary-adrenal axis activity involves regulation by brain regions of the corticolimbic circuit, including excitatory inputs from the amygdala and inhibitory inputs from the prefrontal cortex (PFC) and hippocampus (Howland et al., 2017). Investigations in stress-exposed GF and colonized animals reveal altered gene expression across the corticolimbic circuit (Heijtz et al., 2011; Stilling et al., 2015; Hoban et al., 2016; Gur et al., 2017; Jašarević et al., 2018; Cowan et al., 2019; Provensi et al., 2019), including reductions in the neuronal growth and survival-promoting molecule, brain-derived neurotrophic factor (BDNF) (Bercik et al., 2011; Heijtz et al., 2011; De Palma et al., 2015; Gur et al., 2017; Provensi et al., 2019), and the GR transcription factor, nerve growth factor-inducible protein A (NGFI-A) (Heijtz et al., 2011). These changes in gene expression are accompanied by significant shifts in neuronal activity and myelin-associated gene expression in the PFC of male mice (Hoban et al., 2016). Altered myelination levels and myelin-associated gene expression have been reported in numerous neuropsychiatric disorders, highlighting the significance of such changes on the brain and behavior (Lee, 2009). Interestingly, colonization of GF animals at weaning restored transcriptome levels, but not protein levels, in both sexes (Hoban et al., 2016), supporting the notion that a critical window exists for early-life microbial colonization. Alongside accelerated involvement of prelimbic regions such as the PFC (Cowan et al., 2019), increased anxiety-like behavior (De Palma et al., 2015; Gur et al., 2017, 2019), and alterations in cognition in stress-exposed animals (De Palma et al., 2015; Zeng et al., 2015), it is clear that a dynamic relationship exists between colonization status and neurodevelopmental trajectories.

Ecological changes in the gut microbiota following stress paradigms include shifts in alpha-diversity (i.e., the average number of species in the gut), relative abundance, and specific taxa being reported across numerous studies. However, inconsistencies in these shifts are common, likely due to differences in the degree and timing of stress imparted during stress paradigms, rodent strains, and animal housing conditions. Commonly observed trends include decreased alpha-diversity and shifts in the relative abundance of the dominant phyla, Bacteroidetes and Firmicutes, with increases in potentially pathogenic and facultative anaerobic Proteobacteria (Bailey et al., 2011; Pusceddu et al., 2015; Bharwani et al., 2016; El Aidy et al., 2017; Gur et al., 2017; Moussaoui et al., 2017; Geng et al., 2020). These compositional changes are consistent with literature describing numerous gastrointestinal and neuropsychiatric disease states (Jiang et al., 2015; Vogt et al., 2017; Alam et al., 2020), suggesting stress may have profound effects on gut microbial ecosystems. At lower classification levels, shifts in the abundance of taxa belonging to the bacterial families *Lachnospiraceae* and *Ruminococcaceae* are widely reported, often decreasing following a stressor (Bailey et al., 2011; El Aidy et al., 2017; Moussaoui et al., 2017; Tsilimigras et al., 2018; Bassett et al., 2019; Provensi et al., 2019; Geng et al., 2020). Taxa belonging to these families are considered part of the core microbiota (Falony et al., 2016), found in abundance in term infants (Fouhy et al., 2019) and healthy individuals in large population studies (Falony et al., 2016), suggesting decreases could be indicative of abnormal patterns of microbial succession. Additionally, reports of stress being associated with enrichment of hydrogen sulfide-producing, *Desulfovibrio* (Moussaoui et al., 2017; Bassett et al., 2019; Geng et al., 2020), reductions in the mucus-resident, *Akkermansia* (Bharwani et al., 2016; Moussaoui et al., 2017), and inconsistent shifts in *Rikenellaceae* (Pusceddu et al., 2015; Gur et al., 2017; Jašarević et al., 2017; Tsilimigras et al., 2018) are common in stress literature. *Desulfovibrio* is associated with inflammation due to its role in sulfur metabolism (Carbonero et al., 2012) and is often elevated in ulcerative colitis (Rowan et al., 2010), whereas *Akkermansia* is associated with decreases in inflammation (Zhai et al., 2019), and reductions in its abundance are linked to increased risk for diabetes and obesity (Everard et al., 2013; Earley et al., 2019). Together, it is evident that stress has the capacity to induce extensive and complex compositional shifts in the gut microbiota, with trends toward ecosystem alterations seen in disease states.

**Box 1. Translatability of Stress-Microbiome Animal Study Findings to Humans.**

While animal studies have revealed a causal relationship between the early-life gut microbiome and prenatal or postnatal stress exposures, the extrapolation of these findings to the mother-infant relationship and lifelong health trajectories in humans is constrained by the available data in humans and by the greater complexity of the human situation. Distinct differences exist between the study designs and data obtained in animal versus human studies, and while animal models are a valuable tool to establish proof of principle and examine relationships in the absence of confounding factors, they do not comprehensively reflect human data. At this time, human data exploring this subject area are limited and narrow in scope. Furthermore, it must be recognized that stress-microbiome associations in humans are embedded within social, institutional, cultural and economic structures that inform and shape the nature of these associations. For conclusive statements on the relationship between prenatal or postnatal stress, the infant microbiome, and stress response programming to be made in humans, comprehensive cohort studies including a broad array of factors that may contribute to or be protective against prenatal or postnatal stress and microbiome alterations are essential, such as paternal stress, caregiving practices (e.g., breastfeeding), family dynamics, social environment, and economic status.

While the above studies examined microbiota changes following early-life stress paradigms, evidence of prenatal stress having lasting effects on offspring has also been demonstrated. *Lactobacillus*, one of the dominant taxa in the healthy vaginal microbiota that colonizes infants during birth (Palmer et al., 2007; Dominguez-Bello et al., 2010; Reyman et al., 2019), is decreased in the vaginal microbiota of pregnant rodents (Jašarević et al., 2015, 2018), monkeys (Bailey et al., 2004), and humans (Zijlmans et al., 2015) exposed to gestational stress. This reduction in abundance is subsequently reported in offspring, with microbial community composition deviations from non-stressed controls persisting into adulthood (Golubeva et al., 2015; Jašarević et al., 2015). Significant sex-dependent changes in community composition have also been reported, particularly in male offspring (Gur et al., 2017, 2019; Jašarević et al., 2017). In line with previous findings (Moussaoui et al., 2017; Bassett et al., 2019), enrichment of *Desulfovibrio* has been observed in prenatally stressed female offspring at weaning, alongside the mucus-degrader, *Mucispirillum*, and *Odoribacter* (Jašarević et al., 2017), all of which are associated with colitis (Rooks et al., 2014). Utilizing microbial transplant methods in offspring from control and stressed dams, (Jašarević et al., 2018) found the maternal vaginal microbiome of unstressed dams was unable to rescue prenatal insults to the microbiome of offspring of stressed dams, with these insults being partially mediated by the maternal vaginal microbiome. These findings highlight the importance of prenatal stress exposures, indicating prenatal considerations should be included in the early-life critical window for gut microbial colonization. While shifts in neurodevelopmental and neuropsychiatric trajectories have long been associated with maternal prenatal stress (O’Donnell et al., 2009; Kim et al., 2015), implications for the microbiome in this relationship have only recently been considered.

Given the gut microbiome plays an essential role in gastrointestinal integrity and homeostasis, it is not surprising that early-life stress has also been associated with altered metabolic profiles (Jašarević et al., 2015; Bharwani et al., 2016; Provensi et al., 2019) and increased intestinal permeability (Ait-Belgnaoui et al., 2012; Moussaoui et al., 2017; Jašarević et al., 2018; van de Wouw et al., 2018). These alterations can have detrimental effects on the brain, behavior, and inflammation (Kelly et al., 2015; Witt et al., 2019). As previously mentioned, SCFAs are microbially derived metabolites that are involved in gut-brain communication, mood regulation, and are essential for gut epithelial integrity (Tan et al., 2014; Dalile et al., 2019). In stressed animals, decreases in SCFA profiles have been observed, alongside reduced metabolic activity of pathways involved in SCFA, tyrosine, and tryptophan metabolism and biosynthesis in males (Bharwani et al., 2016; Provensi et al., 2019). Given butyrate is known to have antidepressant effects (Valvassori et al., 2014; Sun et al., 2016), and tyrosine and tryptophan-derived metabolites are precursors for a number of neurotransmitters (O’Mahony et al., 2017; Roager and Licht, 2018), these metabolic shifts have the potential to negatively impact mood, behavior, and brain functioning. Increased intestinal permeability is highly relevant to these outcomes, as resultant inflammatory cascades have been shown to be enriched in the hypothalamus, leading to central neuroinflammation and stress axis activation (Ait-Belgnaoui et al., 2012). Stress exposures in early-life, therefore, have dynamic effects on the gut microbiome, metabolism, intestinal integrity, brain development, and behavior. Emerging evidence suggests these effects are sex-dependent, however, comprehensive research examining stress and microbiome alterations in both sexes is needed to fully understand this relationship.

## Consequences of Stress Axis Alterations on Health & Disease

Inappropriate activation or dysregulation of the HPA axis is involved in numerous pathophysiological outcomes, with many of these processes beginning very early in life. This is in line with the developmental origins hypothesis, which proposes that early-life exposures have extraordinary potency to alter developmental processes and set the stage for disease development in adolescence and adulthood (Gluckman et al., 2005; Waterland and Michels, 2007). Therefore, it is not surprising that parallels exist between the early-life critical windows for the development of major physiological systems, such as the immune and nervous systems, and the establishment of the gut microbiome (Borre et al., 2014; Gensollen et al., 2016; Farzi et al., 2018). A complex interplay exists between these systems, with microbial signals increasingly recognized as important modulators involved in their developmental trajectories, and intricately linked with the stress axis (Farzi et al., 2018; Stiemsma and Michels, 2018). Inter-individual differences in gut microbial community composition (Human Microbiome Project Consortium, 2012) and the variety of factors influencing stress reactivity, amplify the challenge of uncovering causality to this relationship in humans (Box 1). Nonetheless, evidence from animal models has revealed gut microbial colonization is causally implicated in the developmental programming of the stress axis (Foster et al., 2017; Farzi et al., 2018).

Excessive or prolonged stressor exposure during critical developmental windows may lead to chronically hyper- or hypoactive stress and immune responses, altering the homeodynamic state to one that underpins pathophysiological outcomes, such as chronic low-grade inflammation or immunosuppression, respectively (Raison and Miller, 2003; Agorastos et al., 2018). Hyperactivation of the HPA axis results in increased release of GCs (Aguilera and Liu, 2012), with GR insensitivities and/or decreased expression to negative feedback mechanisms prolonging this systemic response (Di et al., 2017). Associated health outcomes range from neuropsychiatric conditions such as depression (Iob et al., 2019), panic disorders (Abelson et al., 2007), and obsessive-compulsive disorder (Gustafsson et al., 2008) to those affecting other body systems such as diabetes mellitus (Prpić-Križevac et al., 2012) and hyperthyroidism (Johnson et al., 2005). In contrast, hypoactivation of the HPA axis is related to disease states such as fibromyalgia (Ciaramella et al., 2017), chronic fatigue syndrome (Van Den Eede et al., 2007), and post-traumatic stress disorder (PTSD) (Morris et al., 2012). Interestingly, PTSD has been associated with reductions in the bacterial phyla Actinobacteria and Verrucomicrobia, specifically *Akkermansia muciniphila* (Hemmings et al., 2017), which were also less abundant in infants whose mothers experienced significant prenatal stress (Zijlmans et al., 2015). Despite differential exposures leading to stress axis dysregulation, the ensuing health consequences exhibit striking similarities, with neuropsychiatric disorders and chronic immune-mediated diseases emerging as common outcomes (Danese et al., 2007; Kuhlman et al., 2017; Agorastos et al., 2018).

Adaptations to stress exposures have historically been viewed as having evolutionary value, as they enable the organism to resist selective pressures (Vanbesien-Mailliot et al., 2007). However, modern day consequences of adaptive mechanisms in response to early-life stress can be particularly detrimental to brain development (Pechtel and Pizzagalli, 2011; Hambrick et al., 2019). Under the influence of significant early-life stress, maturation of the human brain and underlying neuronal circuitry is thought to occur more rapidly, in a period when slower developmental processes are favorable for optimal brain development (Tottenham et al., 2010; Whittle et al., 2013). This forms the basis of the stress acceleration hypothesis, which proposes a reprioritization of developmental strategies to more rapidly achieve adult-like functioning in stress and fear-related brain circuitry when under the influence of early-life stress (Callaghan and Tottenham, 2016). This accelerated maturation has been linked to both structural and functional changes in the brain (Lebel et al., 2016), particularly in corticolimbic regions such as the hippocampus and amygdala (Dannlowski et al., 2012; Teicher et al., 2016), and connectivity between these regions and the prefrontal cortex (Hay et al., 2020). Given the abundant expression of GRs in the hippocampus and its role in cognition, and the central role of the amygdala in stress responsivity, it is not surprising that stress-related insults associate most strongly with these regions (Dannlowski et al., 2012; Swartz et al., 2015). As a consequence of this rapid maturation, early-life stress is strongly associated with the development of neuropsychiatric morbidities in adolescence and adulthood (McLaughlin et al., 2010), with females more frequently being affected (Becker et al., 2007).

Together, it is clear that early-life stress exposures have broad-ranging effects on major body systems, with chronic dysregulation of stress and immune responses emerging as major themes underlying morbidity development (Cohen et al., 2012). Disruption of developmental programming by stress exposure has both central and peripheral effects at molecular, structural, and functional levels (Agorastos et al., 2018), with stress, the gut microbiome, and the development of the immune system all implicated in these complex processes (Foster et al., 2017; Dzidic et al., 2018). As a consequence, it is plausible that alterations in stress axis activity, immune responses, or microbial colonization patterns in the first years of life alone may be sufficient to induce global dysregulation in these systems and increase susceptibility to later life disease development. However, animal model evidence suggests these effects may be reversible. Environmental enrichment (McCreary and Metz, 2016; Dandi et al., 2018), sensitive caregiving (Grant et al., 2009; Thomas et al., 2017), and exposure to beneficial microbes and their by-products (Sudo et al., 2004; Ait-Belgnaoui et al., 2012; Pusceddu et al., 2015; van de Wouw et al., 2018; Cowan et al., 2019; Neufeld et al., 2019; Provensi et al., 2019) during early-life may help prevent the development of adverse behavioral, cognitive, physiological, and neurodevelopmental health outcomes linked to high levels of stress exposure. Further, new understandings of gut microbiota alterations with stress exposure highlight the need to target the bidirectional aspects of this relationship to enable more comprehensive or alternative therapeutic approaches to be developed.

## Microbiome-Related Approaches to Ameliorating Stress Axis Dysregulation

From a microbial perspective, therapeutically targeting the microbiome to recover negative developmental effects from early-life stress is an emerging research area (Table 1). Animal models have primarily focused on supplementation with probiotics, prebiotics, or polyunsaturated fatty acids (PUFAs), but SCFAs have also shown promise for decreasing HPA axis hyperactivity and intestinal permeability in male animals (van de Wouw et al., 2018). The probiotics, *Lactobacillus rhamnosus* (various strains) and *Lactobacillus farciminis*, and prebiotics, polydextrose (PDX) and galacto-oligosaccharide (GOS), have beneficial effects following stress insults, including reducing HPA axis reactivity to stress, CRH expression, and intestinal permeability in females; increasing HPA axis negative feedback inhibition through increased expression of GR, MR and CRH receptors; and ameliorating anxiety-like behavior and gene expression alterations in corticolimbic brain regions in males (Ait-Belgnaoui et al., 2012; Cowan et al., 2019; Neufeld et al., 2019). *L. farciminis* also prevented stress-mediated neuroinflammation in females (Ait-Belgnaoui et al., 2012), whereas PDX prevented accelerated involvement of prelimbic brain regions in fear regulation, and normalized male behavior (Cowan et al., 2019). PUFA supplementation appears to predominantly benefit microbiome-related factors in both sexes, including restoring the Bacteroidetes:Firmicutes ratio and SCFA profiles following stress exposure, and prevents such alterations when administered prior to stress (Pusceddu et al., 2015; Provensi et al., 2019). High dose PUFA supplementation in females reduced Proteobacteria abundance and was associated with increased butyrate-producing bacteria, *Butyrivibrio* (Pusceddu et al., 2015), both of which represent shifts toward healthier microbial communities (Flint et al., 2012; Human Microbiome Project Consortium, 2012). While these preclinical findings are promising for the amelioration of stress-related insults, additional research is required to determine if these benefits will transpire comparably in the clinic.

## Conclusion

The neuroendocrine stress response is a highly conserved physiological system that has persisted in its role as a regulator of homeostasis from the earliest evolved vertebrates to modern humans, highlighting its fundamental role in health maintenance and survival. Developmentally, the plasticity of the stress axis across individuals’ functions to enable appropriate responses to different environments and stressors. In parallel, vertebrates house diverse and abundant microbial communities at multiple body sites, which are known to play an integral role in healthy development. It is thought that these microbes have become an essential environmental exposure informing our developmental trajectories and the programming of major body systems, particularly during the critical window of early life. Compelling evidence of an intricate link between stress and the microbiome has emerged from both experimental animal models and clinical studies examining the relationship between both prenatal and postnatal stress and the composition of the microbiota in mothers and their offspring. This relationship may have profound implications on pathophysiological outcomes, given stress response dysregulation is highly correlated with morbidities emerging in adolescence and adulthood, with these outcomes being sex-dependent.

As of now, the ecological processes that govern the conserved patterns of microbial colonization early in life are only partly understood. Research efforts to further discern these processes will better inform the design of ecologically framed approaches to remediate microbiome perturbations during infancy, including their relationship with stress axes. Further, scientists have relied on animal behavioral readouts to interpret stress, when in reality, this is typically an internal response that is often of strategic advantage not to convey to the external world. As a consequence, uncertainties regarding the translatability of understandings generated from animal models are evident, highlighting the need for human cohort studies examining the interplay between the neuroendocrine stress response, immune system, and microbial colonization in early-life across both sexes, as well as the influence of this interplay on the developmental programming that underpins disease outcomes.

Given the complex relationship between the development of the immune system, neuroendocrine stress response, and the gut microbiome in early-life, future research examining the relationship between caregiving practices, such as skin-to-skin contact (e.g., kangaroo care), and stress-immune-microbiome interactions may provide valuable insights into strategies to reduce or prevent undesirable health outcomes in infants exposed to significant early-life stress, or microbial alterations. The influence of kangaroo care and tactile stimulation on the microbiota and stress have been evaluated independently, with evidence of benefits for these practices on desired health outcomes (Harrison, 2001; Feldman et al., 2010; Hendricks-Muñoz et al., 2015). However, integrative studies are yet to be performed. While the interplay between stress and the microbiome has only recently been revealed, it is probable that targeting the microbiome in stress-immune pathologies will transform therapeutic approaches in the future.

## Author Contributions

VO and EM equally conceptualized, researched, and authored the manuscript with editorial assistance from GG and M-CA. All authors approved the final manuscript.

## Conflict of Interest

The authors declare that the research was conducted in the absence of any commercial or financial relationships that could be construed as a potential conflict of interest.
